# Parietal Activation Associated With Target-Directed Right Hand Movement Is Lateralized by Mirror Feedback to the Ipsilateral Hemisphere

**DOI:** 10.3389/fnhum.2018.00531

**Published:** 2019-01-09

**Authors:** Thushini Manuweera, Mathew Yarossi, Sergei Adamovich, Eugene Tunik

**Affiliations:** ^1^Rutgers School of Graduate Studies, Rutgers University, Newark, NJ, United States; ^2^Department of Biomedical Engineering, New Jersey Institute of Technology, Newark, NJ, United States; ^3^Department of Physical Therapy, Movement, and Rehabilitation Sciences, Northeastern University, Boston, MA, United States; ^4^Department of Electrical and Computer Engineering, College of Engineering, Northeastern University, Boston, MA, United States

**Keywords:** mirror feedback, target, motor control, fMRI, virtual reality, visuomotor integration

## Abstract

Current research shows promise in restoring impaired hand function after stroke with the help of Mirror Visual Feedback (MVF), putatively by facilitating activation of sensorimotor areas of the brain ipsilateral to the moving limb. However, the MVF related clinical effects show variability across studies. MVF tasks that have been used place varying amounts of visuomotor demand on one’s ability to complete the task. Therefore, we ask here whether varying visuomotor demand during MVF may translate to differences in brain activation patterns. If so, we argue that this may provide a mechanistic explanation for variable clinical effects. To address this, we used functional magnetic resonance imaging (fMRI) to investigate the interaction of target directed movement and MVF on the activation of, and functional connectivity between, regions within the visuomotor network. In an event-related fMRI design, twenty healthy subjects performed finger flexion movements using their dominant right hand, with feedback presented in a virtual reality (VR) environment. Visual feedback was presented in real time VR as either veridical feedback with and without a target (VT+ and VT-, respectively), or MVF with and without a target (MT+ and MT-, respectively). fMRI contrasts revealed predominantly activation in the ipsilateral intraparietal sulcus for the main effect of MVF and bilateral superior parietal activation for the main effect of target. Importantly, we noted significant and robust activation lateralized to the ipsilateral parietal cortex alone in the MT+ contrast with respect to the other conditions. This suggests that combining MVF with targeted movements performed using the right hand may redirect enhanced bilateral parietal activation due to target presentation to the ipsilateral cortex. Moreover, functional connectivity analysis revealed that the interaction between the ipsilateral parietal lobe and the motor cortex was significantly greater during target-directed movements with mirror feedback compared to veridical feedback. These findings provide a normative basis to investigate the integrity of these networks in patient populations. Identification of the brain regions involved in target directed movement with MVF in stroke may have important implications for optimal delivery of MVF based therapy.

## Introduction

Mirror visual feedback (MVF), which involves observing the mirror reflection of moving one’s hand to give the visual impression of opposite hand movement, has been shown to alter brain activation when compared to direct observation of the moving hand. Studies that assessed changes in cortical activity associated with MVF training using Transcranial Magnetic Stimulation (TMS) have shown an increase in the magnitude of motor evoked potentials, a measure of corticospinal excitability, ipsilateral to the moving hand both online (Garry et al., 2005; Funase et al., 2007; Kumru et al., 2016) and offline (Nojima et al., 2012; Yarossi et al., 2017). Investigations using fMRI to study MVF have described the activation of a network of sensorimotor areas ipsilateral to the moving hand in both healthy (Hamzei et al., 2012; Wang et al., 2013; Fritzsch et al., 2014; Rjosk et al., 2017) and stroke participants (Michielsen et al., 2011; Saleh et al., 2014; Saleh et al., 2017). The ability to activate the ipsilateral hemisphere provides a basis for MVF as a viable treatment option for patients with unilateral deficits and limited movement of the impaired hand. Importantly, the change in ipsilesional sensorimotor activation observed after MVF-based training seems to relate to functional gains (Dohle et al., 2009; Thieme et al., 2013) in stroke patients.

However, careful examination of numerous investigations reveals wide variation in the observed neurophysiological response (Deconinck et al., 2015) and clinical outcomes (Veerbeek et al., 2014) associated with MVF-based training. One possible source of variability may be the task performed with MVF. Previously used tasks vary substantially in the requirement for visuomotor integration for successful task completion, and tasks that require less visuomotor integration may have less pronounced MVF-elicited effects. We have specifically shown this to be true in the case of MVF effects on corticospinal excitability in healthy individuals. Yarossi et al. (2017) showed that the addition of a target-directed movement (requiring visuomotor integration) to MVF training resulted in greater modulation of corticospinal excitability, compared to MVF training that did not require the subject to perform movements to a visually defined target. That finding was discussed in the context of the effect that the action observation network, comprised of bilateral visual and motor areas, may play in mediating MVF. However, that study could not directly examine the involvement of the action observation network, because TMS was used to assess M1 excitability only. The present investigation builds on this knowledge-base by using fMRI to test the dependence of visuomotor task-specificity paired with MVF, on a fronto-parietal network associated with action observation.

The involvement of the parietal cortex in execution and observation of visually guided target-directed movements of the hand has been investigated extensively (Hamilton and Grafton, 2006; Tunik et al., 2007; Hamilton and Grafton, 2008; Cavina-Pratesi et al., 2017). This body of work highlights the activation of the anterior intraparietal sulcus during observation of goal-directed actions, which is significantly less responsive for observation of non-goal directed actions (Buccino et al., 2001). Importantly, some studies have reported bilateral parietal activation for unimanual tasks that require successful ongoing computation and transformation of spatial coordinates (Grefkes et al., 2004), and processing movement error when reaching toward targets (Diedrichsen et al., 2005). It is therefore plausible that execution of target-directed movements may activate parietal regions bilaterally in a way that facilitates the modulatory activity between the parietal areas and motor cortices.

The aim of the current study was to investigate whether the interaction of MVF and visuomotor demand leads to stronger activation of, and functional connectivity between, brain areas of the visuomotor network in the ipsilateral hemisphere in young healthy adults. Based on previous investigations which separately tested the effects of MVF and target-directed movement in healthy individuals (Hamzei et al., 2012; Yarossi et al., 2017), we hypothesize that target-directed movements combined with MVF will be associated with stronger ipsilateral fronto-parietal activation than MVF or target-directed movements alone. The results of this study provide important information about the neural mechanisms involved in processing MVF, and whether target-directed actions are necessary to engage those networks.

## Materials and Methods

### Participants

Twenty healthy, right-handed (Oldfield, 1971) adults (8F, mean age 25.6 ± 3.9 years) participated following institutionally-approved informed consent. All subjects were free of neurological or psychiatric conditions, history of head trauma resulting in loss of consciousness, cognitive impairments or dementia, and met all safety requirements for MRI. Subjects with orthopedic pathology of the upper limb or visual impairments that interfered with the task were excluded from the study.

### Setup

**FIGURE 1:**
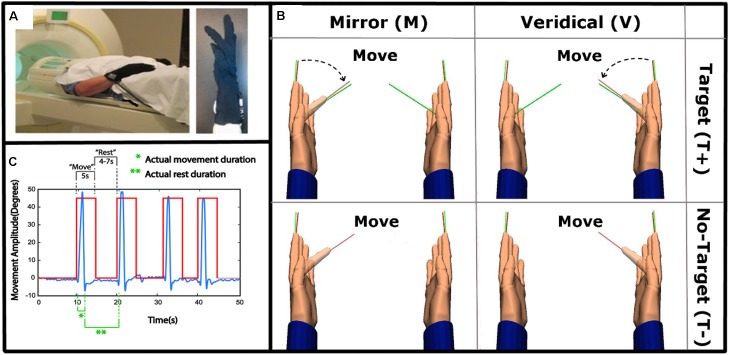
Experimental set-up and task. **(A)** Left: The experimental set-up with subject wearing data gloves bilaterally. Right: All movements were performed with the right, dominant, hand. **(B)** 2 × 2 study design, randomly alternating between Mirror with Target (MT+), Veridical (no-Mirror) with Target (VT+), Mirror with no-Target (MT-) and Veridical with no-Target (VT-) conditions throughout the event-related fMRI run. **(C)** A sample kinematic data trace (blue) spanning four different trials, with the box-car function of the cues (red) overlaid. Green notations demark movement and rest times that were modeled in the GLM.

Participants wore MRI compatible fiber optic recording gloves (Fifth Dimension Technologies Inc, Pretoria, South Africa) on both hands (Figure 1A). Sensors embedded in the gloves measured the metacarpophalangeal and proximal interphalangeal finger joint angles. The gloves were interfaced with a virtual reality (VR) environment, using Virtools software (Dassault Systemes, Vélizy-Villacoublay, France) that was viewed in the scanner on the presentation screen. The VR representation of the hands were shown in the first-person view, i.e., left and right are the same as of the subject’s. The real time joint angle data streaming from the gloves actuated a corresponding motion of the virtual hand, and this data was recorded for statistical analysis of movement kinematics.

### Task and Conditions

The experiment was designed to identify brain regions activated during target-directed mirror feedback while performing the task using the right hand only. Each condition was repeated eight times in a pseudorandom order within a functional run. Each subject performed 4 runs. The ‘Move’ cue was displayed for 5 s, and the ‘Rest’ cue was displayed for a duration of 4–7 s, randomly varying in length to increase the jitter between trials. Prior to data collection, subjects were familiarized with the task by performing each condition until they executed the task correctly according to the instructions.

Each trial began with the hand fully open, and aligned with the return line (green line in Figure 1B). Upon a visual ‘Move’ cue, subjects were instructed to flex their finger with the goal of aligning the red line protruding from the index fingertip to either a green target line (target-directed conditions) or to the perceived middle of the range of movement (no target conditions) (Figure 1B). The display of the move cue was the same across all conditions. Subjects were asked to make discrete, fast, and accurate movement, and to avoid making online corrections. After briefly pausing at their terminal finger angle, subjects were to return to the initial position and await the next trial (Yarossi et al., 2017). To test how MVF feedback interacts with the presence of target-directed movements, four conditions were performed in a 2 × 2 factorial design:

• Mirror Target condition (MT+): The left virtual hand was actuated by the subject’s moving right hand. Green target line present.
• Veridical Target condition (VT+): The right virtual hand was actuated by the subject’s moving right hand. Green target line present.
• Mirror No-Target condition (MT-): The left virtual hand was actuated by the subject’s moving right hand. No target line present.
• Veridical No-Target condition (VT-): The right virtual hand was actuated by the subject’s moving right hand. No target line present.

### Movement Kinematics

Kinematic data were analyzed in order to verify that movements were consistent across feedback conditions and runs. Trials with incorrect movements (missed trials or movement corrections) were excluded from the analysis. Consistency of movement was tested using angular velocity and movement amplitude as outcome measures. For each trial, movement onset and offset were defined as the time at which the mean angular velocity of the 4 metacarpophalangeal joints exceeded and then fell below 10% of the mean peak angular velocity respectively. Movement amplitude was quantified as the maximum angular excursion between the onset and offset. Peak angular velocity, as well as movement amplitude, were averaged across trials for each functional imaging run and each condition, and then analyzed using separate two-way repeated-measures analyses of variance (rmANOVA) with factors: Target-directed movement (Target, No-Target), and Feedback type (Veridical, Mirror). Statistical significance was set at *p* < 0.05.

### fMRI Data Acquisition and Analysis

All data were acquired using a 3-T Siemens Magnetom TrioTim syngo MR B17 scanner. The parameters used for high-resolution T1 MPRAGE structural images were: repetition time (TR), 2 s; echo time (TE), 25.6 ms; voxel size, 1 mm^3^; and slice thickness, 1 mm. The parameters used for functional images were: T2^∗^-weighted echo planar imaging sequence; TR, 2 s; TE, 30 ms; field of view, 192 mm; voxel size, 3mm3; number of slices, 40; inter-slice time, 62 ms; and number of volumes, 173. All fMRI data were preprocessed and analyzed using SPM12 (fil.spm@ucl.ac.uk, London) software. The first two scans were acquired in order to account for field inhomogeneities, and were not included in analysis. All functional data were first realigned, then slice time corrected, co-registered to structural space, and then normalized using SPM12 DARTEL toolbox to the standard brain in the Montreal Neurological Institute (MNI) space. All scans were smoothed using an 8 mm Gaussian kernel. General Linear models were created for each individual subject and each experimental condition. Data analysis was conducted at the single-subject (fixed-effects) and group levels (random-effects). Statistical significance for all analyses was set at *P* < 0.05 [cluster-level false discovery rate (FDR) corrected (voxel extent k > 10)]. The following T-contrasts were created for the main effects of Target (Target vs. No Target), Feedback (Mirror vs. Veridical), and Target-directed MVF.

• Contrast 1: Main effect of Target-directed movement,
  (Target[mirror]+Target[veridical])>(No-Target[mirror] +No-Target[veridical])
• Contrast 2: Main effect of Mirror feedback,
  (Target[mirror]+No-Target[mirror])>(Target[veridical] +No-Target[veridical])
• Contrast 3: Effect of target-directed movement with mirror feedback,
  Target[mirror]>(Target[veridical]+No-Target[mirror]+ No-Target [veridical])

### Conjunction Analysis

A conjunction analysis (Friston et al., 1999) was used to identify brain regions commonly activated for the main effect of Target-directed movement (contrast 1) and Mirror feedback (contrast 2). Thereafter, strength of activation was tested for each condition, in the regions identified by the conjunction analysis. For this, a mask was created for the thresholded activation map of the conjunction and beta scores were extracted from each main effect of condition and compared using an ANOVA. Statistical significance was set at FDR corrected *p* < 0.05; with minimal cluster size of *k* = 10. *Post hoc* comparisons were done using Tukey’s honest significant difference test. Effects were considered significant at *p* < 0.05.

### Functional Connectivity

Functional connectivity has been described as the experiment and time-dependent causal influences that one brain region exerts over another (Friston, 1994). In this study, functional connectivity was quantified between the ipsilateral parietal cortex and ipsilateral motor cortex using the generalized psychophysiological interaction (PPI) toolbox for SPM12. PPI analysis estimates which voxels in the brain increase in connectivity with a given seed region of interest during a particular behavioral task (O’Reilly et al., 2012). The seed region for the ipsilateral parietal cortex was defined as the highest activated voxel in contrast 3 (MT+ > VT+MT-VT-). The mean MNI x, y, z coordinates (±1 SD) of the seed was 24 ± 2.4, -60 ± 3.3, 63 ± 2.9, corresponding to the superior parietal lobule (BA7). The seed was located on the medial bank of the intraparietal sulcus (mIPS), corresponding to area IPS3 (Swisher et al., 2007). This region has been shown to respond to visuomotor processing of a target in the contralateral space (Davare et al., 2012).

The functional connectivity map for the seed of interest with the rest of the brain was generated for each subject and each condition. The PPI interactions were then compared using an analysis of variance (ANOVA) testing the effect of interest contrast and a repeated measures analysis of variance (rmANOVA) at group level testing the following contrast:

• Effect of target-directed movement with mirror feedback,
  Target[mirror]PPI>(Target[veridical]PPI+No-Target [mirror]PPI+No-Target[veridical]PPI)

PPI coefficients were extracted from each subject for each condition for the connectivity between the seed region and motor cortex and then compared using a two-way rmANOVA with factors: Target-directed movement (Target, No-Target), and Feedback type (Veridical, Mirror). Statistical significance was set at *p* < 0.05.

## Results

### Kinematics Across Conditions

Hand kinematics were inspected to identify any missed trials and inadvertent or corrective movements. Only 1.15% of all trials were excluded from the analysis. Consistency of hand kinematics across trials was verified using separate two-way rmANOVAs for peak angular velocity and movement amplitude. A significant main effect of Target was present for peak angular velocity [*F*(1,18) = 46.66, *p* < 0.001], which was 49.4 deg/s higher in the No-Target (MT-, VT-; 349.72 ± 138.00) relative to the Target conditions (MT+, VT+; 300.32 ± 118.74). Likewise, a significant main effect of Target was present for movement amplitude [*F*(1,18) = 64.34, *p* < 0.001], which was 8.22 degrees greater in the No-Target (48.96 ± 10.83) relative to the Target conditions (40.74 ± 10.31). No other significant main effects or interactions were noted. As illustrated in Figure 1C, the participants performed fast movements, according to the instructions that lasted about 600–700 ms, even though the move cue was present for 5 s.

### fMRI Analysis

fMRI contrasts were used to understand differences in brain activity for the main effects of Target-directed movement (Contrast 1, T+ > T-), Feedback type (Contrast 2, M > V), and the combination of MVF with target directed movements (Contrast 3, MT+ > VT+MT-VT-).

#### Effect of the Presence of a Target on Brain Activation (T+ > T-)

**FIGURE 2:**
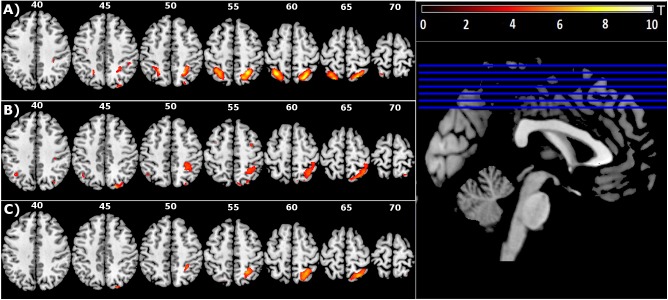
Group level main effect of: **(A)** Target-directed (T+ > T-) movement, **(B)** Mirror feedback (M > V), and **(C)** Mirror feedback combined with a Target-directed movement (MT+ > VT+MT–VT–) (FDR corrected *P* < 0.05; minimal cluster size *k* = 10).

The presence of a target was associated with significantly increased activation in bilateral superior parietal lobes (SPL), with activation in the right hemisphere extending to the precuneus, including IPS4. The activation of IPS4 was observed in both left and right hemispheres (Figure 2A and Table 1).

**Table 1:** Results for all contrasts.

	Region	Side	T Statistic	*P*-value (FDR corrected)	Coordinate (MNI) x, y, z			*k*
Main effect: T+ > T-	Intraparietal sulcus, IPS2	Right	8.79	0.000	21	-66	60	203
	Precuneus	Right	8.71	0.000	24	-57	54	
	Medial intraparietal sulcus, IPS4	Right	7.94	0.008	28	-54	54	
	Superior Parietal, BA7	Left	8.06	0.001	-27	-60	57	171
	Medial intraparietal sulcus, IPS4	Left	5.79	0.017	-27	-55	55	
Main effect: M > V	Precuneus, IPS1	Right	6.17	0.017	21	-81	45	249
	Superior Parietal	Right	5.79	0.017	33	-54	63	
	Anterior intraparietal sulcus, hPGR	Right	4.52	0.025	30	-42	51	
	Inferior Parietal, Supramarginal gyrus	Right	5.59	0.017	27	-48	51	
	Inferior Parietal, Angular gyrus	Left	4.25	0.023	-36	-60	42	20
	Inferior Parietal, Angular gyrus	Left	4.09	0.026	-42	-60	48	
Effect of Mirror feedback w/Target	Medial intraparietal sulcus, IPS3	Right	6.73	0.003	24	-60	63	163
	Medial intraparietal sulcus, IPS4	Right	5.14	0.014	27	-54	54	
	Anterior intraparietal sulcus, hPGR	Right	4.2	0.026	30	-42	51	


*Significant clusters are FDR corrected (*P* < 0.05; extent *k* > 10) at the voxel level. *k*, cluster size in voxels; x, y, z, coordinates (mm) of the peak voxel in the MNI coordinates.*

#### Effect of Mirror Feedback on Brain Activation (M > V)

Mirror feedback was associated with the activation of bilateral superior and inferior parietal lobes and the precuneus of the right hemisphere. The human parietal grasp region (hPGR) (Mruczek et al., 2013) was a noteworthy region activated in the right hemisphere of this contrast. A small but significant cluster of activation was noted in the left inferior parietal cortex (Figure 2B and Table 1).

#### Brain Activation Related to Mirror Feedback During Target-Directed Movement (MT+ > VT+MT-VT-)

The combination of mirror feedback with target-directed movement was associated with significant activation of the right SPL, including areas IPS3, IPS4 and hPGR, both of which were regions separately noted in the G+ > G- contrast and M > V contrast (Figure 2C and Table 1). Note that this parietal activation is ipsilateral to the moving hand.

#### Conjunction Analysis

Conjunction analysis was used to identify brain regions commonly activated for the main effects of Target and Mirror feedback. Beta scores were extracted from brain regions identified from this analysis to test for differences in activation strength.

**FIGURE 3:**
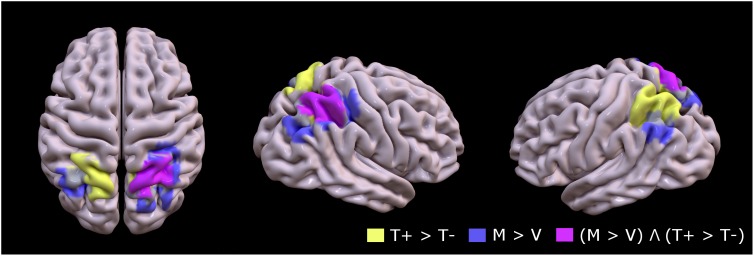
Group mean of contrast 1 (T+ > T–, yellow), contrast 2 (M > V, lavender), and conjunction between contrast 1 and contrast 2 [(M > V) Λ (T+ > T–), pink] to identify topographic overlap between brain regions recruited during motion of the untrained hand with mirror feedback, and areas recruited during movement to targets (FDR corrected *P* < 0.05; minimal cluster size *k* = 10).

Figure 3 (pink overlay) shows the overlapping territory in the right superior parietal (BA7), specifically the medical intraparietal areas, precuneus and inferior parietal areas that were jointly activated in the two contrasts (FDR corrected *P* < 0.05; minimal cluster size *k* = 10).

**FIGURE 4:**
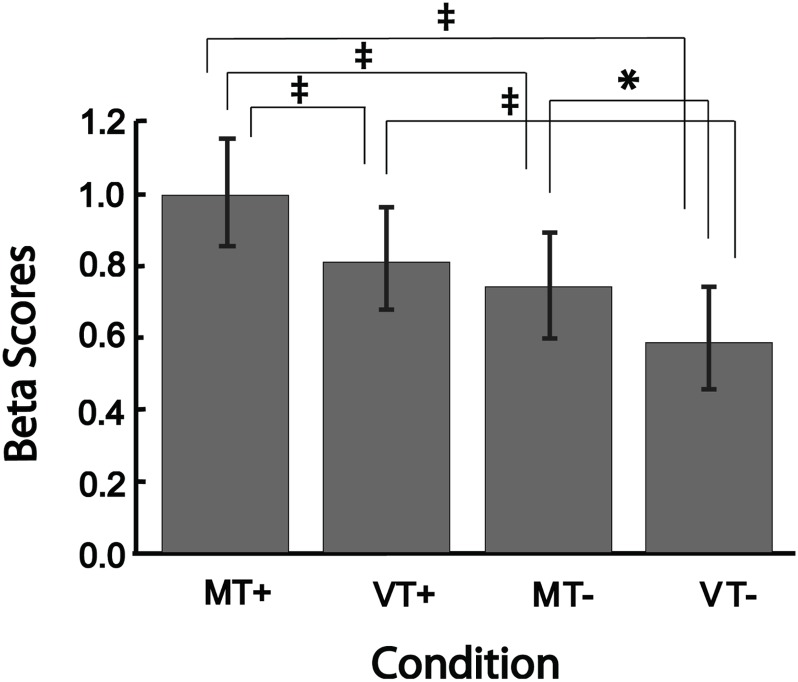
Bar plots showing group level brain activation in each condition (MT+, VT+, MT–, VT–) at the ROI identified from the conjunction analysis. Error bars represent ± 1 SEM. ‡Indicates *p* ≤ 0.002; ^∗^indicates *p* < 0.05. *Post hoc* comparisons were significant (*p* < 0.05) for all comparisons except between VT+ and MT–.

Figure 4 shows the mean beta scores extracted for each condition in the main effect contrast using the conjunction mask. The one-way rmANOVA performed between conditions was significant [*F*(3,15) = 24.29, *p* < 0.001], with Tukey’s *post hoc* comparisons revealing significant differences between MT+ and each of the other three conditions (MT-, VT+, VT-; all *p*-values < 0.002), between VT+ and VT- (*p* < 0.001), and between MT- and VT- (*p* = 0.013). The beta scores of VT+ were not significantly different from the scores of MT-. These results suggest that mirror feedback was associated with significantly stronger activation in the right hemisphere, which was further strengthened when MVF was combined with a target-directed movement.

#### Brain Activation Related to Movement Without a Target

Significant differences in peak angular velocity between with and without target conditions, and the main effect of Target presence on brain activation (contrast 1), warranted the investigation of the absence of target. Brain activation patterns in the No-Target > Target comparison (No-Target[mirror] + No-Target[veridical] > Target[mirror] + Target[veridical]) revealed that movements performed without a visually defined target were associated with significant activation in the left post central gyrus extending to the precentral gyrus, and bilateral insula (Table 2).

**Table 2:** BOLD activation that was greater for movements performed without a target than toward a target.

	Region	Side	T statistic	*P*-value (FDR corrected)	Coordinate (MNI) x, y, z			*k*
Effect of absence of target	Postcentral gyrus	Left	8.79	0.000	-18	-33	78	1120
	Insula	Left	7.80	0.000	-45	-18	12	207
	Insula	Right	4.46	0.008	48	-33	18	242
								


*Significant clusters are FDR corrected (*P* < 0.05; extent *k* > 10) at the voxel level. *k*, cluster size in voxels; x, y, z, coordinates (mm) of the peak voxel in the MNI coordinates.*

#### Functional Connectivity With IPS3 Seed Region

Psychophysiological interaction analysis was used to quantify the functional connectivity between the IPS3 seed region, which was identified from contrast MT+_PPI_ > VT+_PPI_MT-_PPI_VT-_PPI_, and the ipsilateral motor cortex.

**FIGURE 5:**
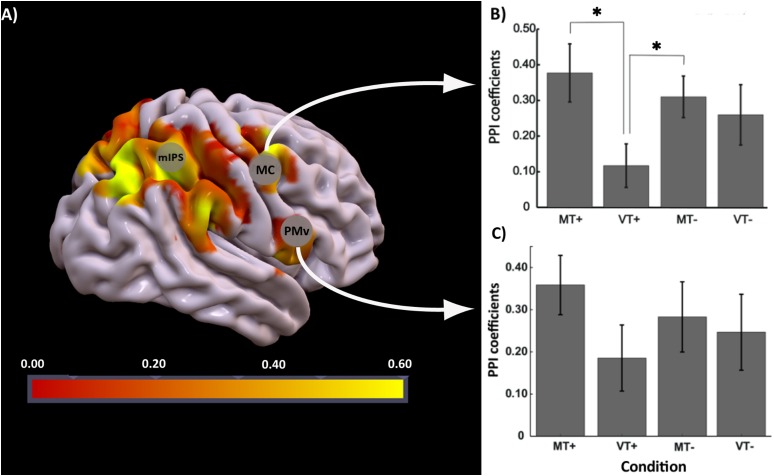
PPI analysis: **(A)** Brain regions activated for the contrast MT+_PPI_ > VT+_PPI_MT–_PPI_VT–_PPI_ with seed region at IPS3, **(B)** bar plots showing group level strength of functional connectivity in each condition (MT+, VT+, MT–, VT–) between IPS3 seed and hand knob of M1 (MC), *post hoc* comparisons were significant (*p* < 0.05) between MT+ and VT+, and VT+ and MT–, **(C)** bar plot showing group level strength of functional connectivity in each condition (MT+, VT+, MT–, VT–) between IPS3 seed and ventral premotor cortex (PMv). Error bars represent ± 1 SEM. ^∗^Indicates *p* < 0.05 significance.

The effect of interest contrast tested the PPI interaction in each condition compared to rest. There was significant activation bilaterally in parietal and motor areas (FDR corrected *p* < 0.05; *k* = 10). A binary mask of these significantly activated brain regions was created and used to identify voxels that were active in contrast MT+_PPI_ > VT+_PPI_MT-_PPI_VT-_PPI_ at *p* < 0.05 uncorrected. The highest activated voxel in the primary motor cortex (BA4) was located at coordinates *x* = 30, *y* = -33, *z* = 60 (MNI) in the medial hand knob. Another cluster with its highest activation at coordinates *x* = 54, *y* = 6, *z* = 9 (MNI) was noted in the ventral-most part of the premotor cortex (PMv). Correlation coefficients representing the strength of the connectivity between the seed in IPS3 and motor regions were extracted for each condition and subject and compared at the group level using a two-way rmANOVA. As shown in Figure 5, statistical analysis revealed a significant main effect of Feedback [*F*(1,18) = 10.36, *p* = 0.002] and a significant Feedback^∗^Target [*F*(1,18) = 4.71, *p* = 0.034] interaction for the connectivity between the seed and the primary motor cortex. Tukey’s *post hoc* comparisons indicated significant differences between MT+ and VT+ (*p* = 0.002), and between VT+ and MT- (*p* = 0.032). No significant effects were noted between conditions for the connectivity between the seed and the premotor cortex. The motor regions showing connectivity to the seed region are associated with hand movements, sensorimotor learning, and sensorimotor integration (Binkofski and Buccino, 2006). The right parietal reach region (PRR) at coordinate *x* = 27, *y* = -75, *z* = 45 belonging to the posterior parietal cortex was also functionally connected to the seed region, though the connectivity coefficients did not reach statistical significance between the MT+ and the other conditions.

## Discussion

In this study, we investigated the response of visuomotor processing regions in the brain to the pairing of MVF with target-directed movements. Participants completed four conditions with either mirror or veridical feedback and either presence or absence of a visual target while performing index finger movements with real time VR feedback. Presence of a Target (contrast 1) increased bilateral parietal activation, and Mirror (contrast 2) resulted in predominately ipsilateral parietal activation. The combination of Target presence and Mirror (contrast 3) resulted in strong activation of ipsilateral parietal areas. A significant difference in the functional connectivity was observed between areas IPS3 and the motor cortex when target-directed movements were performed with MVF compared to veridical.

### Combining MVF With Target-Directed Movement Selectively Increases Ipsilateral Parietal Activation

Brain regions that are activated during both action execution and observation of movement, are known to make up the Action Observation Network (AON), and studies have shown evidence suggesting that this network may be involved in MVF processing (Matthys et al., 2009; Saleh et al., 2014, 2017). Among the areas recruited during action observation, parietal regions are known to be associated with the observation of biological motion (Nelissen et al., 2011; Rizzolatti et al., 2014), which may underlie the activation that we observe in the parietal cortex in this study. It is likely that the activation in these regions are relayed via a dorsomedial pathway from area V6, which encodes for visual motion (Galletti and Fattori, 2018), though it is not entirely surprising that V6 itself was not significantly activated in the subtracted contrast given the fact that visual motion was equally present in all conditions. Actions that require visuomotor processing of a target or a goal (e.g., reaching and grasping) have been associated with activation of the anterior intraparietal sulcus (aIPS, BA7) indicating parietal areas of the AON are also involved in the integration of target goal and action planning (Hamilton and Grafton, 2006; Tunik et al., 2007, 2008). Studies have shown that parietal activation tends to be bilateral for target directed reaching/pointing tasks (Grefkes et al., 2004; Diedrichsen et al., 2005; Imamizu and Kawato, 2008; Cavina-Pratesi et al., 2017). These studies, and ours, have in common the demand for online visuomotor processing and spatial transformations to enable successful and precise attainment of the target. Importantly, our data support previous findings, showing the target present condition was associated with stronger bilateral activation primarily in the SPLs that extended toward the anterior portion of the intraparietal sulci, than in target absent conditions (contrast 1).

Mirror feedback was also associated with significant bilateral parietal activation, in agreement with prior studies testing MVF-related activation in healthy individuals that have shown activation in parietal areas (Hamzei et al., 2012; Wang et al., 2013; Fritzsch et al., 2014). The extent of parietal activation with MVF was notably larger in the ipsilateral than the contralateral hemisphere (contrast 2). Lateralization of parietal activation to the ipsilateral hemisphere during MVF compared to direct visual feedback has also been previously shown for movements performed using the right hand (Mehnert et al., 2013; Fritzsch et al., 2014).

Key to understanding the effects of combining target-directed movement and MVF, are the results from the conjunction analysis, which revealed that the medial intraparietal region of the ipsilateral SPL was jointly activated during both target-directed movements and MVF. It has been shown that the SPL is integrally involved in visual spatial attention (Muri et al., 1996; Donner et al., 2000; Silver et al., 2005; Offen et al., 2010). Interestingly, visuomotor-based SPL activation seems to be tuned to what is happening in the contralateral visual field (Kertzman et al., 1997; Medendorp et al., 2003). It is also important to note that specifically the area corresponding to the human aIPS, also known as the human parietal grasp region (hPGR) was activated in the contrast that tested the presence of mirror feedback, and prior studies in both macaque (Sakata et al., 1995) and humans (Shikata et al., 2001; Grefkes and Fink, 2005; Konen et al., 2013) have shown that this particular area is strongly activated when processing grasping movements and, discriminating orientation of visual stimuli. In addition, the anterior part of the medial intraparietal sulcus corresponding to the IPS4 area was activated in contrasts that tested the presence of a goal. This area of the intraparietal sulcus (IPS4) is known to be involved in execution of grasping movements (Konen et al., 2013) and implementing the direction vector of visually guided movements performed toward contralateral targets (Davare et al., 2012), which explains why this particular area would be activated when target-directed movements are combined with mirror feedback. It is therefore possible that MVF acts to facilitate activation in the above-named regions of SPL ipsilateral to the moving hand, since the mirrored hand is located in the opposite visual field. Most noteworthy is that the ability of MVF to achieve this effect is conditional on whether the movement is target-directed. This latter finding underscores the critical need to enforce a strict visuomotor demand on the subject, in order to activate the desired SPL.

### Role of Ipsilateral Parietal Cortex in Mediating MVF to Ipsilateral Motor Areas

Empirical data lend support for the modulatory influence that parietal cortex has over M1. Among other evidence, dual-coil paired-pulse TMS experiments demonstrate that a conditioning stimulus applied to anterior parts of the intraparietal sulcus and BA5 has a facilitory influence over M1 during planning of reaching movements toward visually defined targets (Koch et al., 2008; Ziluk et al., 2010). Our functional connectivity analysis suggests that the connectivity between IPS3and the ipsilateral motor cortex significantly increased for the target-directed movements performed with Mirror, relative to Veridical, feedback. This effect is consistent with our previous work in stroke subjects (Saleh et al., 2014) which demonstrated that MVF can increase the modulatory effective connectivity from bilateral parietal regions to ipsilesional M1 (Saleh et al., 2017). However, in contrast to our previous results in stroke patients showing modest MVF-related activation of M1, significant MVF-related ipsilateral M1 activation was not found in the current study with healthy subjects. Indeed most studies in healthy populations do not show significant MVF-related activation in the ipsilateral M1 (Hamzei et al., 2012; Mehnert et al., 2013; Wang et al., 2013; Fritzsch et al., 2014). One possible reason for this discrepancy is that ipsilateral motor cortex in a neurologically damaged system may be more responsive to the sensory attributes of MVF feedback, because of the need to re-learn motor functions (Rizzolatti and Craighero, 2004; Avenanti et al., 2013). Such activation of the motor system may build more slowly in health individuals, such as when learning is allowed to accrue. We have demonstrated such slower accrual of M1 activation in healthy individuals in our lab in the past (Bagce et al., 2013; Yarossi et al., 2017). Although it remains to be directly tested, it may be that the sensory aspects of MVF (without learning) may be sufficient to activate the motor system of patients, but may be limited to activating the predominantly sensorimotor integration regions of the parietal cortex in healthy participants. Finally, compensatory activation patterns resulting from stroke (Grefkes et al., 2008; Pundik et al., 2015) may also explain the discrepancy of motor cortex activation between the two population groups. Although non-significant, the region closely corresponding to the PRR showed greater connectivity to the seed region when mirror feedback was combined with target directed movements. In other work, this region of the brain has been implicated in encoding information related to the intention of making movement to a particular spatial location (Connolly et al., 2003). The role in processing spatial information, when performing intended limb movements, may explain why this region was functionally connected to the seed for target directed conditions compared to the other conditions.

### Activation Was Not Likely Due to Movement Vigor

Kinematic results did not reveal a significant main effect of Feedback type or Target × Feedback interaction, but did reveal a significant main effect of Target presence, where the movement speed was greater when movements were performed without a target. Previous investigations indicate M1 and somatosensory cortex (S1) activation scales linearly with increased movement rate for repetitive finger tapping (Blinkenberg et al., 1996; Rao et al., 1996; Sadato et al., 1997; Wexler et al., 1997; Lutz et al., 2005), however, less is known about discrete movement tasks such as the one performed in this study. Inference about discrete movements can be made from findings indicating increased motor cortex, supplementary motor cortex and basal ganglia activation for discrete force production tasks (Sergio et al., 2005). Increased activation of areas of the postcentral gyrus with faster movement in the no target condition (relative to with a target) appear to be in line with these previous results. However, differences in movement speed or amplitude have not, to our knowledge, been associated with changes in parietal activation and therefore do not explain our primary finding of increased bilateral parietal activation with the addition of a target. Furthermore, since the parietal regions actually showed the inverse effect (e.g., being more strongly activated in the condition for which movements were slower), it is unlikely that this activation could be explained by a vigor effect. The alternate view is that the effect is due to greater visuomotor processing that was likely required in the target present, compared to target absent, conditions.

### Study Limitations

We only tested the right hand. Prior MVF work has shown differences in lateralization of activation depending on the hand used to perform the task (Wang et al., 2013; Fritzsch et al., 2014). Although these studies do not show a consensus in areas differently activated depending on which hand is used, nevertheless this suggests that there is a possibility that effects observed in the current study could change with the use of the non-dominant left hand. We did not measure the potential impact of motor imagery. However, given that visual information was necessary to perform guided movements, particularly in the target conditions, effects of motor imagery on the current results are unlikely. Future work could explicitly control for this possibility by adding an imagery condition. To make this paradigm more broadly relevant to the stroke population, it is important to also incorporate a finger extension task, which may be well-aligned to the rehabilitation needs that often face patients. Although the task used was a finger flexion task, we speculate that our findings should generalize across muscles.

## Conclusion

This study highlights the importance of target-directed movement to activate the ipsilateral parietal cortex with MVF. Understanding how target-directed feedback can influence activity of the parietal areas and the parietal-M1 connectivity during movement with MVF in healthy individuals provides a basis for examining the efficacy of targeted-directed MVF for enhancing activation of this network and functional gains in stroke.

## Ethics Statement

This study was carried out in accordance with the recommendations and approval of the Institutional Review Board of Rutgers Biomedical Health Sciences. All subjects gave written informed consent in accordance with the Declaration of Helsinki.

## Author Contributions

TM, MY, ET, and SA contributed to the conception and design of the research, analyzed and interpreted the data, and edited and revised the manuscript. TM performed the experiments. TM and ET prepared the figures and drafted the manuscript. All authors approved the final version of this manuscript.

## Conflict of Interest Statement

The authors declare that the research was conducted in the absence of any commercial or financial relationships that could be construed as a potential conflict of interest.
